# A Radiomics-Clinical Model Predicts Overall Survival of Non-Small Cell Lung Cancer Patients Treated with Immunotherapy: A Multicenter Study

**DOI:** 10.3390/cancers15153829

**Published:** 2023-07-28

**Authors:** Sevinj Yolchuyeva, Elena Giacomazzi, Marion Tonneau, Leyla Ebrahimpour, Fabien C. Lamaze, Michele Orain, François Coulombe, Julie Malo, Wiam Belkaid, Bertrand Routy, Philippe Joubert, Venkata S. K. Manem

**Affiliations:** 1 Department of Mathematics and Computer Science, Université du Québec à Trois Rivières, Trois-Rivières, QC G8Z 4M3, Canada; 2 Quebec Heart & Lung Institute Research Center, Québec City, QC G1V 4G5, Canadamichele.orain@criucpq.ulaval.ca (M.O.);; 3 Centre de Recherche du Centre Hospitalier Universitaire de Montréal, Montréal, QC H2X 0A9, Canada; 4 Université de Médecine de Lille—Université Henri Warembourg, 59020 Lille, France; 5 Department of Physics, Engineering Physics and Optics, Laval University, Quebec City, QC G1V 4G5, Canada; 6 Department of Molecular Biology, Medical Biochemistry and Pathology, Laval University, Québec City, QC G1V 0A6, Canada

**Keywords:** immunotherapy, non-small cell lung cancer, machine learning, overall survival, radiomics

## Abstract

**Simple Summary:**

In the recent years, immune checkpoint inhibitors (ICIs) have significantly modified non-small cell lung cancer (NSCLC) treatment by providing new therapeutic avenues with superior efficacy and improved tolerability over traditional cytotoxic therapies. The immune activation can lead to durable clinical responses and prolonged survival in some, but not in all of the treated NSCLC patients. Thus, there is an unmet clinical need to identify patients who are most likely to have short-term overall survival against prolonged overall survival (OS). These patients can thus be spared from potential toxicities as well as from the financial burden arising from these immune therapies. Most importantly, early prediction of these patients may allow the clinicians to choose more aggressive or effective treatment options early on, to extend the overall survival of patients. Through this study, we developed parsimonious survival risk models incorporating clinical data and imaging profiles of NSCLC patients treated with immunotherapy. These results may enable the clinicians to design more effective therapeutic regimens or modify treatment strategies for the group of short-term survivors.

**Abstract:**

Background: Immune checkpoint inhibitors (ICIs) are a great breakthrough in cancer treatments and provide improved long-term survival in a subset of non-small cell lung cancer (NSCLC) patients. However, prognostic and predictive biomarkers of immunotherapy still remain an unmet clinical need. In this work, we aim to leverage imaging data and clinical variables to develop survival risk models among advanced NSCLC patients treated with immunotherapy. Methods: This retrospective study includes a total of 385 patients from two institutions who were treated with ICIs. Radiomics features extracted from pretreatment CT scans were used to build predictive models. The objectives were to predict overall survival (OS) along with building a classifier for short- and long-term survival groups. We employed the XGBoost learning method to build radiomics and integrated clinical-radiomics predictive models. Feature selection and model building were developed and validated on a multicenter cohort. Results: We developed parsimonious models that were associated with OS and a classifier for short- and long-term survivor groups. The concordance indices (C-index) of the radiomics model were 0.61 and 0.57 to predict OS in the discovery and validation cohorts, respectively. While the area under the curve (AUC) values of the radiomic models for short- and long-term groups were found to be 0.65 and 0.58 in the discovery and validation cohorts. The accuracy of the combined radiomics-clinical model resulted in 0.63 and 0.62 to predict OS and in 0.77 and 0.62 to classify the survival groups in the discovery and validation cohorts, respectively. Conclusions: We developed and validated novel radiomics and integrated radiomics-clinical survival models among NSCLC patients treated with ICIs. This model has important translational implications, which can be used to identify a subset of patients who are not likely to benefit from immunotherapy. The developed imaging biomarkers may allow early prediction of low-group survivors, though additional validation of these radiomics models is warranted.

## 1. Introduction

Lung cancer is the leading cause of cancer-related deaths worldwide [1]. Non-small cell lung cancer (NSCLC) accounts for approximately 80% of all lung cancer cases, and the majority of these are diagnosed at an advanced stage [2,3]. For many years, alkylating agents have been the main therapeutic option for patients diagnosed with advanced non-small cell lung cancer, conferring a median overall survival (OS) of around six months [4,5]. Immune checkpoint inhibitors (ICIs) have revolutionized the treatment paradigm for patients diagnosed with a broad range of malignancies, including advanced NSCLC [5]. ICIs have now become the standard of care and can be administered either alone or in combination with chemotherapy as the first-line therapy for treatment-naïve patients and as the second-line therapy for patients who do not respond to chemotherapy [6,7]. These molecules block an escape mechanism developed by the tumor to inactivate and avoid the immune cells of the host system.

Two landmark randomized trials in chemotherapy-resistant NSCLC patients demonstrated a benefit in OS in the nivolumab (anti-PD-1) arm compared to docetaxel standard second-line chemotherapy [8]. In the checkmate clinical trial, patients diagnosed with non-squamous NSCLC who were treated with nivolumab had a longer OS than those treated with docetaxel [8]. In addition, the results of the Keynote trial, a phase II/III study that enrolled patients with advanced NSCLC (with a PD-L1 value of at least 1%), demonstrated a survival benefit of pembrolizumab compared with docetaxel [9,10]. A long-term survival benefit was also observed in patients (with PD-L1 ≥ 50%). Despite these results, primary resistance to ICI remains unpredictable and exceeds 60%, while secondary resistance rates approach 100%, highlighting the need for novel biomarkers independent of histology to be able to predict the OS of NSCLC patients treated with these expensive therapies.

ICIs have been shown to have more durable responses than the existing cytotoxic therapies among advanced NSCLC patients. Despite durable responses to immunotherapy agents, most patients show primary or acquired resistance. Therefore, there has been increasing attention directed toward understanding the resistance mechanisms for improving the long-term clinical outcomes of patients receiving these ICIs. Although it is known that immunotherapy can bring long-term survival to patients with advanced NSCLC, it is important to know how long in general the immune-related treatment can bring significant OS benefit to advanced NSCLC patients. Furthermore, the decreased OS in advanced NSCLC patients may represent that a subgroup of patients either do not respond to immunotherapy or might potentially have hyper-progression. Therefore, it is crucial to build novel biomarkers that can distinguish patients who will have a long-term benefit from ICIs in terms of OS from those who are likely to progress despite therapy with short-term OS.

‘Radiomics’ is an emerging field that leverages CT scans and aims to analyze the extracted tumor features in a high-throughput manner. The field of lung cancer greatly benefits from radiomics, which leverages imaging data to improve the clinical management of cancer diagnosis, prognosis, and the development of effective therapies. In order to gain insight into tumor characteristics and treatment effects, radiomics involves the extraction and analysis of quantitative aspects from medical images such as computed tomography (CT) scans. The prognostic value of radiomics-based analysis of lung cancer imaging has been demonstrated [11,12]. Overall survival, disease-free survival, and recurrence risk can all be predicted using radiomics models by quantifying a variety of imaging variables like tumor size, shape, texture, and vascular properties. Clinicians can use these data to create individualized treatment regimens and decide how much follow-up is necessary. Furthermore, these quantitative features are extracted from a region of interest (ROI) of medical scans to build biologically and clinically relevant models for predicting patient-specific endpoints, such as OS, progression-free survival (PFS), response to various therapeutic interventions, etc. [13]. One of the earliest works using radiomics was carried out by Aerts et al., who built a prognostic signature that was validated for both lung and head & neck cancers [14]. The authors extracted a total of 440 radiomic features quantifying tumor intensity, shape, and texture from CT images and developed a Cox model to predict the survival of patients. Along these lines, several studies in the literature have attempted to build radiomics-based signatures in the context of immunotherapy. In a study by Zerunian et al., the authors developed a model using the CT-scan-derived features to predict OS and progression-free survival in 21 patients with advanced NSCLC who had been treated with first-line pembrolizumab [15]. In another study by Braghetto et al., the authors compared handcrafted radiomics- and deep-learning-based approaches on the publicly available lung cancer dataset for the prediction of two-year OS in NSCLC patients [16]. Studies in literature have shown the utility of radiomics as a promising imaging-based biomarker in the management of NSCLC receiving ICIs. However, none of these studies have attempted to build survival risk predictive models integrating radiomics and clinical features from a multi-institutional perspective for the prediction of OS as well as for the prediction of short- and long-term time-to-survival events in patients treated with immunotherapy.

In this study, we aim to develop radiomics and integrative radiomics-clinical predictive models to predict OS as a continuous value and short-term and long-term survivor groups in the context of OS. The non-invasive imaging biomarkers developed in this study can potentially identify low-group survivors early on, which could help the clinicians to design and/or modify (effective) therapeutic interventions for this cohort of patients. Furthermore, in the present work, we used two independent cohorts from different institutions to develop and validate the data-driven imaging-based biomarkers, which will have greater generalizability and clinical relevance.

## 2. Materials & Methods

### 2.1. Description of Cohorts

This study includes a retrospective cohort of samples retrieved from two thoracic oncology reference academic centers, the Institut Universitaire de Cardiologie et de Pneumologie de Québec (Quebec Heart and Lung Institute, IUCPQ) and the Centre Hospitalier Universitaire de Montréal (CHUM). These two centers are known for the diagnosis and clinical management of patients with lung cancers. The study has been approved by the respective institutional ethics committees (MP-10-2020-3397/CÉR CHUM: 19.390). We conducted a retrospective study to evaluate the OS among patients presented with advanced NSCLC and treated with ICIs. The samples used for this study were part of the Quebec Respiratory Health Network Tissue Bank (https://rsr-qc.ca/biobanque/) at the IUCPQ—The data was accessed from the patients treated after 1 January 2015. To be eligible for the study, patients must have signed a written informed consent form, been presented with a histologically advanced stage of NSCLC, and been treated with ICIs. At least one breath-hold chest pre-ICI CT scan (3 months before the administration) and one post-treatment CT scan were available to assess disease progression following the initiation of the treatment (using RECIST 1.1 criteria). The dataset from the two centers included 385 patients in total, out of which 223 patients were part of the discovery cohort (CHUM) and 162 patients from the in-house validation cohort (IUCPQ).

### 2.2. PD-L1 Assessment

PD-1 is expressed on the surface of activated T-cells and down-regulates T-cell activity upon binding to its ligands: PD-L1 and PD-L2 [17,18]. As part of standard patient management following lung cancer diagnosis, PD-L1-positive tumor cells (or tumor proportion score (TPS)) were assessed using immunohistochemistry (IHC) labeling (Dako Autostainer) with the 22C3 clone (pharmDx kit). The TPS was then used to measure PD-L1 expression, which is expressed as the proportion of tumor cells with positive membranous staining ranging from 0 to 100%. Each tumor was then grouped based on the clinical cut-off value for PD-L1 TPS, which was 1%, 1–49%, or ≥50% [19].

### 2.3. Overall Survival Assessment

OS is defined as the time from the date of diagnosis either to the date of death due to any cause or to the date of censoring at the last time the subject was known to be alive [20]. In this study, we focused on two objectives: (i) to predict the clinical endpoint, namely, the continuous value, OS, and (ii) to build a classifier for predicting the short- and long-term survivor groups of NSCLC patients treated with ICIs. OS being a continuous value, we considered the first objective as a regression-based task. It should be noted that in the next few sections, we termed it as OS-R. To build the classifier for the second objective, we split both CHUM and IUCPQ datasets into three quantiles [21]. The first and last quantiles were defined as the short- and long-term survivor groups, respectively. Therefore, this was considered as a binary classification task.

### 2.4. CT Scan Annotation

Each primary lesion was manually annotated by a radiation oncologist or radiologist on the de-identified CT scans that were taken 3 months prior to the administration of immunotherapy. To extract the radiomics features, the region of interest (ROI) was determined through the following procedure: (i) after CT scan alignment, mathematical morphology-based denoising was used for chest isolation, which was followed by chest segmentation using the connected regions; (ii) the lung contour was roughly segmented, and the pulmonary parenchyma was refined after the skin boundary detection; and (iii) the lung nodule ROI was identified independent of the size, position, and any spreading near or through the pleura based on the relative symmetry of the lung.

### 2.5. Extraction of Radiomics Features and Pre-Processing

We used PyRadiomics (v 3.0.1) [22], an open-source Python package for the extraction of handcrafted radiomics features from the region of interest of CT scan data. In a conventional pipeline using PyRadiomics, the radiomics features were extracted and grouped into four main categories:

(i) Tumor intensity-based features: Intensity-based features are first-order statistical features that quantify the tumor’s intensity characteristics. They are derived from the distribution of intensities within the region of interest (ROI) and include mean, median, and mode, as well as measures of the distribution’s symmetry and heterogeneity, such as percentiles, skewness, kurtosis, and entropy. The mathematics behind first-order features includes simple statistical calculations that capture information about the distribution of pixel intensities within the image. For instance, the mean, which represents the average intensity value of all the pixels in the image, is calculated by summing up all the pixel intensities and dividing it by the total number of pixels. Entropy, on the other hand, measures the randomness of the pixel intensity distribution and can be calculated based on the probabilities of different intensity values occurring within the image. These features provide information about the overall distribution of intensities within the ROI and can help distinguish between different histologies. However, they may not capture more complex features, such as spatial variations or patterns, that may be present in the image.

(ii) Shape-based features: Shape-based or morphological features are computed in both 2D and 3D views and include a subset of features that describe the shape and structure of the image, such as volume, surface area, compactness, and sphericity. The mathematics behind shape-based features involves various measurements and calculations related to the volumes of the ROI. For instance, Volume, which represents the total number of voxels enclosed by an ROI, can be computed by summing up the voxel volumes, which is possible through multiplying the number of voxels in the ROI by the volume of a single voxel. Sphericity, which is another commonly used shape-based feature, describes how closely the ROI resembles a perfect sphere. It can be computed as the ratio of the surface area of a sphere with the same volume as ROI to the actual surface area of ROI. These features can be particularly useful in identifying and characterizing complex structures, such as tumors or lesions, that may be difficult to visualize with other imaging techniques.

(iii) Texture-based features: Texture-based or second-order features consider the statistical dependencies and spatial relationships between neighboring voxels and can be computed using various techniques such as gray level co-occurrence matrix (GLCM), gray level run length matrix (GLRLM), gray level size zone matrix (GLSZM), gray level distance zone matrix (GLDZM), and neighborhood gray tone difference matrix (NGTDM). For instance, GLCM quantifies the occurrence of pairs of pixel intensities at various spatial distances and orientations within the ROI and represents them in a matrix. Several statistical measures can be calculated from the GLCM matrix, including Contrast, which represents the local intensity variations between neighboring pixels, or Energy, which is a measure of homogeneous patterns in the ROI and represents the sum of squared elements in the GLCM. A greater Energy implies that there are more instances of intensity value pairs in the ROI that neighbor each other at higher frequencies. These features capture more complex patterns and spatial variations that may not be apparent from other features and can provide additional information about tissue heterogeneity, composition, and organization.

(iv) Filter-based features: Filter-based radiomic features are features that can be extracted from derived images using filters such as Wavelet, Laplacian of Gaussian (LoG), Square, SquareRoot, Logarithm, Exponential, and Gradient. In the current study, the Wavelet filter was applied to texture- and intensity-based features using wavelet transformation. When applied to intensity-based features, wavelet analysis can provide additional insights into the distribution and statistical properties of pixel intensities.

Before applying the feature selection and machine learning methods, various pre-processing steps were performed. The complete dataset was standardized using sklearn’s Standard-Scaler [20]. The scaler is fit to the data and afterwards, transformed to mean, is zero and standard deviation one.

### 2.6. Feature Selection and Machine Learning Methods

To build imaging-based prediction models for both objectives, we used the ReliefF-based feature selection method [23,24], which is commonly used to identify the most relevant features [25,26]. It is particularly useful for dealing with high-dimensional datasets, where there may be a large number of irrelevant or redundant features. This method was based on the original Relief-based algorithm [23]. The main idea behind ReliefF was to evaluate the relevance of each feature in a dataset by estimating how well it distinguishes between instances that are similar and instances that are different.

We then employed the widely known XGBoost machine learning algorithm to achieve both objectives, i.e., to predict OS-R and OS short- and long-term survivor groups, respectively. The XGBoost was first introduced by Chen et al. in [27] and stands for eXtreme Gradient Boosting, which describes an ensemble approach based on the gradient boosting algorithm [28,29]. The gradient boosting algorithm was further developed to improve the performance of the predictive model as well as to decrease the computational time through parallelization. Extreme refers to its intensive performance improvements in comparison to the original gradient boosting algorithm. Ensemble methods like gradient boosting build stronger models by combining multiple weak models, most often the decision trees. In XGBoost, added models correct the errors of existing models to improve the overall performance. By adding new models, the gradient descent algorithm is used to minimize the loss. XGBoost is easily applicable with very low computational costs (for an ensemble method) and is widely known to show good predictive performance [30,31]. We used XGBoost for both regression and classification-based approaches.

### 2.7. Analysis Framework and Model Overview

**Figure 1:**
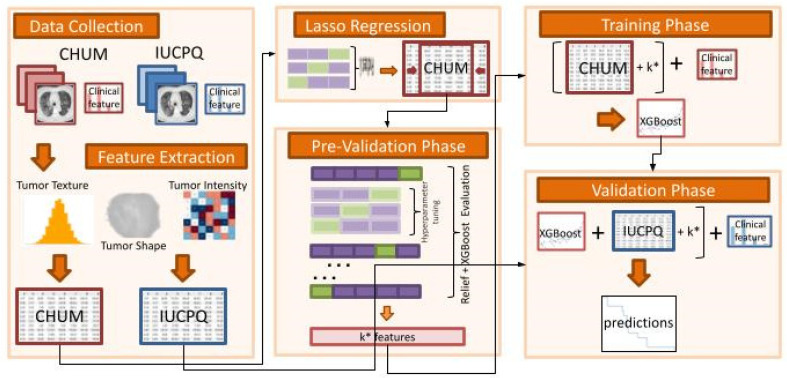
Analysis pipeline of the study. We first performed the pre-validation analysis of radiomics (and integrated radiomics-clinical model) predictors for OS and short- and long-term survivor groups using the cross-validation framework in the discovery cohort (CHUM). The predictors using the full training set were then built and evaluated for their performance in a fully independent validation dataset (IUCPQ). (* denotes the best number of features).

The analysis pipeline considering the feature selection method and machine learning approach for developing the radiomics model and the integrated radiomics-clinical model is illustrated in Figure 1.

We divided the entire procedure into six steps:

- Data Collection:
  ○ The IUCPQ and CHUM centers served as the study’s two academic reference centers for thoracic oncology, and the study included a retrospective cohort of samples obtained from those institutions.
- Feature Extraction:
  ○ To extract handcrafted radiomics features from the region of interest of CT scan data for CHUM and IUCPQ, we utilized the PyRadiomics Python package. There were 851 radiomics features.
  ○ We performed the data standardization, i.e., all variables were required to have a mean of zero, and a standard deviation of one was done for both cohorts.
- Lasso Regression:
  ○ As a first step feature selection method, we used Lasso regression for the OS-R task and Lasso Logistic regression with penalty ‘l1′ for the OS short-term and long-term survival binary task. The Lasso and Lasso Logistic regressions were implemented on the whole training cohort, with the main feature selection Relief and the XGBoost machine learning method applied separately in the next step. However, to find the best *alpha* for Lasso regression and *C* for Logistic regression, we applied grid-search with 5-fold cross validation. Then, we removed all features with coefficients equal to zero after training Lasso on the whole CHUM cohort.
- Pre-Validation Phase:
  ○ Using the resulting features (with those that were retained after the LASSO), we incrementally selected features by employing the ReliefF feature selection method and XGBoost machine learning approach. This was performed in the pre-validation phase on the discovery dataset, which consisted of a 5-fold cross-validation with 10-times repetitions. At this stage, ReliefF feature selection method was employed for each fold.
  ○ The highest C-index for OS-R and AUC for OS short-term and long-term survival was used to determine the best number of features.
  ○ The hyperparameter tuning of XGBoost was also performed through a cross-validation using the GridSearchCV class of sklearn. The ReliefF feature selection was implemented using the Rebate [32] open-source library, and the XGBoost regressor and classifier were implemented using the XGBoost [33] library in Python (scikit-learn version 1.0.2, Python version 3.9.13, XGBoost version 1.6.2 and Skrebate version 0.62) [34].
- Training Phase (without clinical data):
  ○ The XGBoost model with the best hyperparameter was trained on the discovery cohort (CHUM) with the best features based on the pre-validation phase.
- Validation Phase (without clinical data):
  ○ The final model was validated on the independent cohort with best features (IUCPQ).

### 2.8. Radiomics-Clinical Model

After selecting the best radiomics features that yielded the highest concordance index, or AUC, we included only the statistically significant clinical features with the outcome and assessed the model’s performance on the discovery dataset. This was performed in the pre-validation phase on the discovery dataset, which consisted of a 5-fold cross-validation with 10-time repetitions. In other words, the radiomics-clinical model was constructed using both radiomic features and significant clinical features to predict the continuous value of OS as well as to build the classifier for OS short- and long-term survival groups. The model was then validated on the independent cohort.

### 2.9. Model Performance

For evaluation metrics, we used the concordance index (C-index) and AUC for OS-R and OS short- and long-term survival tasks, respectively. The C-index metric is defined as the probability that two variables will rank a random pair of samples in the same order and is a generalization of the area under the ROC curve. A random predictor would result in an index of 0.5, while a perfect predictor yields an index of 1 [35]. AUC stands for “area under the ROC curve” and measures the entire two-dimensional area underneath the entire ROC curve from (0,0) to (1,1).

## 3. Results

### 3.1. Patient Characteristics

**Figure 2:**
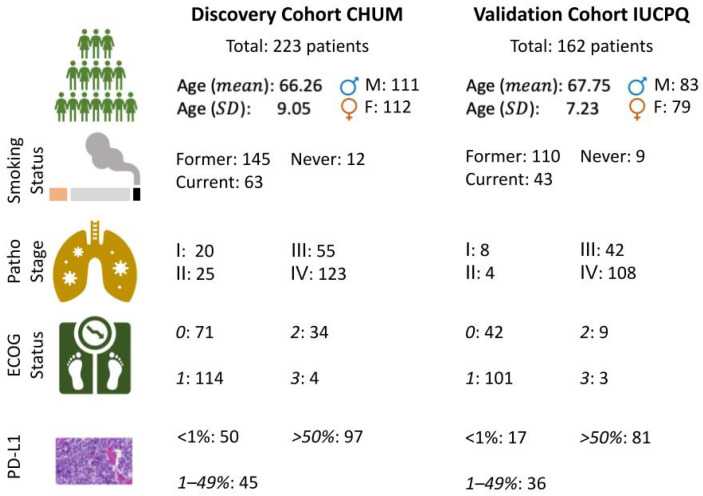
Patient characteristics in the discovery and validation cohorts.

The clinical characteristics of the two cohorts used in the current study are presented in Figure 2. The discovery cohort CHUM consists of 223 patients with an average age of 66.26 years (±9.05). Within the smoking category, 66% of the patients were former smokers, and 28% of them still smoke. In addition, 55% of the patients have a pathologic stage IV tumor, 24% have a stage III tumor, and the remaining 10% have a stage I or stage II tumor. The ECOG performance status [36] is a value on a scale from 0 to 5, where 0 describes a person who is fully active and has the same physical condition as before the disease, and 5 declares the death of a person. It is evaluated by the oncologist/pulmonologist at the time of diagnosis. A clinical research nurse or other professional extracted the data from the patients’ hospital records. The majority of patients in the cohorts are labeled with an ECOG performance status of 1. The validation cohort, IUCPQ, consists of 162 patients with an average age of 67.75 years (±7.23). Among these patients, 67% of them were former smokers, and 26% still smoke. The majority of the patients in this cohort present with pathological stage IV, with 65%. Moreover, the number of patients with PD-L1 as a clinical endpoint is presented in the last row of Figure 2. Overall, 97 out of 223 patients and 81 out of 162 patients have a PD-L1 value ‘≥50%’ in the discovery and validation cohorts, respectively. The number of patients whose PD-L1 value is ‘<1%’ is 50 and 17 in the discovery and validation cohorts, respectively. The range of OS values was 0.3–67.3 months in the CHUM dataset and 0.4–59.4 months in the IUCPQ dataset. For the OS short-term and long-term survival task, the number of samples in the discovery and validation cohort is 149 and 109 respectively. For the discovery cohort, the number of months for short-term survival and long-term survival changed in the range of 0.3–9.2 and 19.9–67.3. And for the validation cohort, the range was 0.4–12.8 and 25.4–59.4 for the short-term and long-term survival groups.

### 3.2. Univariate Analysis of Clinical Variables

The clinical variables used in this study were the following: age, sex, ECOG status, and smoking status of the patients. To evaluate the association between OS and clinical factors, we performed the univariate analyses (using Pearson correlation for continuous value and logistic regression for categorical value) independently. The *p*-values resulting from the univariate analysis are presented in Table 1. From our analysis, we found ECOG performance status to be a clinically significant variable associated with the OS as a continuous value as well as for the OS short- and long-term survivor groups.

### 3.3. Radiomics Features

To build the predictive non-invasive radiomics biomarkers, a total of 851 radiomics features were extracted from the segmented tumor areas of the pre-ICI CT scans of two different cohorts. The CHUM cohort was utilized for feature selection and regression/classification training, while the IUCPQ cohort was used to evaluate the model performance for OS and OS short- and long-term survivor groups.

### 3.4. Univariate Analysis of Radiomics Features

**Figure 3:**
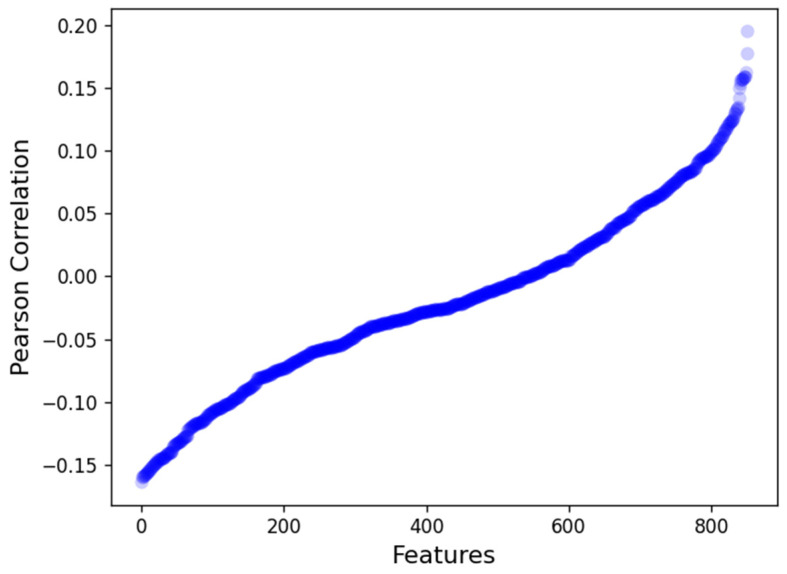
Pearson correlation of radiomics features with the overall survival in the discovery cohort.

We then computed the association between each of the radiomics features and OS using the Pearson correlation in the discovery cohort, which is presented in Figure 3. The maximum and minimum correlation values were found to be 0.20 and −0.17, respectively. We found the maximum negative correlation and positive correlation values with OS to be from the wavelet-based features group with *r* = −0.164 and r = 0.19. Overall, we found that 307 features had a positive correlation with OS, while 544 features were found to have a negative correlation with OS.

### 3.5. Model Evaluation

**Figure 4:**
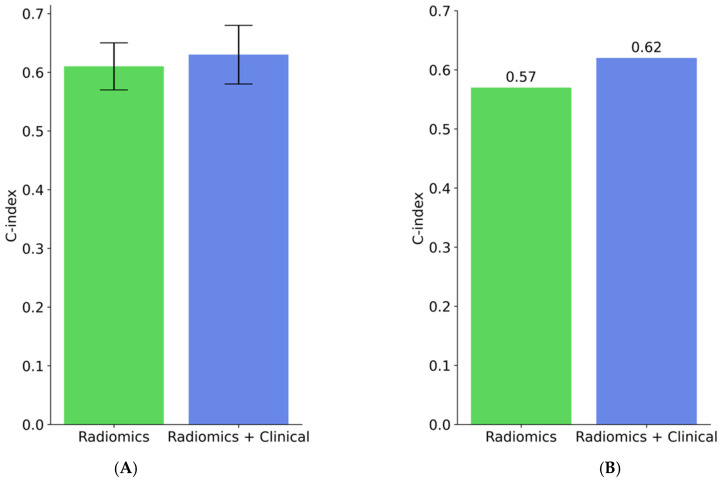
Performance of radiomics and the ensemble radiomics-clinical models to predict the continuous value of OS. (**A**) C-index values on the discovery cohort and (**B**) C-index values on the validation cohort.

In the following section, we will present the results of the two objectives discussed in this study. Figure 4 depicts the performance of the radiomics and radiomics-clinical models for OS-R as concordance indices—discovery cohort in Figure 4, panel A and validation cohort in Figure 4, panel B. The radiomics model yielded around 0.61 in the discovery set and 0.57 in the validation cohort. With the ensemble radiomics-clinical model, the concordance index was slightly improved from 0.61 to 0.63 on the discovery cohort and from 0.57 to 0.62 on the validation cohort.

**Figure 5:**
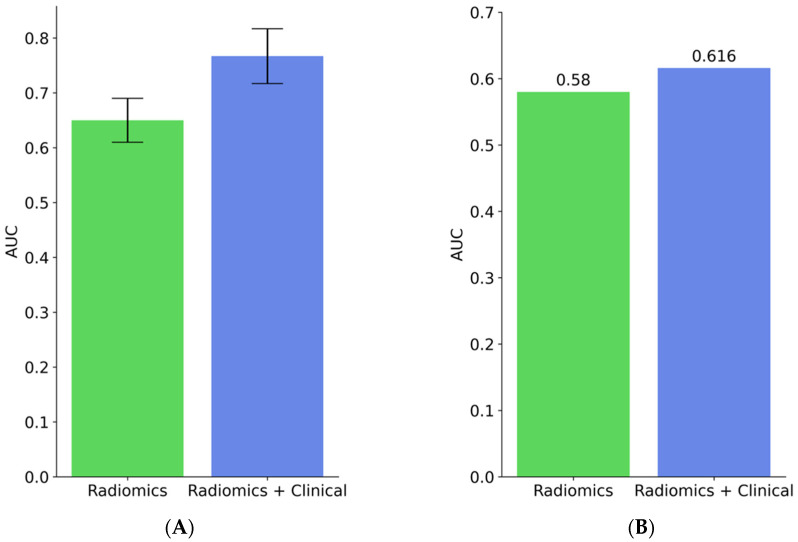
Performance of radiomics and the ensemble radiomics-clinical models to predict the OS short- and long-term survivor groups. (**A**) AUC scores in the discovery cohort and (**B**) AUC scores in the validation cohort.

To build a radiomics-clinical predictive model, we incorporated only the statistically significant clinical features (Table 1) with the radiomics features. Figure 5 presents the performance of the radiomics and radiomics-clinical models for OS short-term and long-term survival groups. Panels (A) and (B) present the AUC in the discovery and validation cohorts, respectively in Figure 5.

The radiomics model resulted in 0.64 and 0.58 in the discovery and validation cohorts. We found that with the ensemble radiomics-clinical model, the AUC values were improved in both the pre-validation and validation phases. Specifically, the AUC in the discovery cohort was significantly improved from 0.65 to 0.767 and in the validation cohort from 0.58 to 0.616. In addition to the above described predictive models and feature selection strategies, we also implemented a combination of several feature selection methods, such as Surf and MultiSurf [24], and machine learning approaches, such as Linear Regression, Random Forest Regressor, Support Vector Machines Regressor, AdaBoost Regressor, and Decision Tree Regressor, for the OS-R task. However, we found that XGBoost combined with ReliefF feature selection method resulted in a better performance when compared to other machine learning-based models.

## 4. Discussion

For many years, cytotoxic chemotherapeutic compounds have been the mainstay for patients presented with advanced non-small cell lung cancer, which accorded an OS of around six months [4]. ICIs represent the standard of care for patients with several types of advanced stage cancers, including NSCLC patients, resulting in a paradigm shift of therapeutic interventions [5]. The immune activation can lead to durable clinical responses and prolonged survival in some, but not all, of the treated patients. Thus, there is an unmet clinical need to identify patients who are most likely to have short-term survival against prolonged OS. These patients can thus be spared from potential toxicities as well as from the financial burden arising from these immune therapies. Furthermore, early prediction of these patients may allow the clinicians to choose more aggressive or effective treatment options early on to prolong the overall survival of patients. While there have been efforts that are focused on building exploratory data-driven biomarkers in the context of immunotherapy and other therapies leveraging several data modalities, there remains a need to identify non-invasive ways to build prognostic and predictive models for immunotherapies.

The advent of radiomics presented a novel non-invasive strategy to build survival risk models as well as to monitor response to various treatments, including ICIs [13,14,37,38]. Despite the growing field of biomarker research in immuno-oncology, none of these prognostic and predictive biomarkers have been translated to routine clinical use in the context of predicting patient outcomes to ICIs in a metastatic setting or to survival. Moreover, none of them have been successfully validated in multicenter settings. Efforts are currently underway to develop more efficient and accurate biomarkers for survival models as well as to improve the patient stratification with ICIs.

Based on this premise, in the current study, we attempted to build biomarkers to achieve two objectives: (i) to predict the continuous value, OS, and (ii) to build a classifier for short-term and long-term survivor groups based on OS. For this purpose, we leveraged clinical and radiomics profiles from two institutions. The pre-ICI CT scan data was used to extract 851 radiomics features with the PyRadiomics platform, which were then used to build the predictive models. There are four clinical variables being examined in this project: the patients’ age, gender, ECOG status, and smoking history. First, we conducted univariate analyses (Pearson correlation for continuous value and logistic regression for categorical value) to examine how different clinical variables were associated with OS. From our analysis, we found ECOG performance status to be a clinically significant variable associated with the OS as a continuous value as well as with the OS short- and long-term survivor groups. Our findings demonstrated that a model that incorporates both radiomics and clinical data (ECOG) outperforms a model that only uses radiomics alone. By incorporating clinical data along with radiomic features, a clinical-radiomics model can provide a more comprehensive picture of the patient’s condition. As a next step, we utilized the ReliefF feature selection method for the regression and classification tasks. The XGBoost machine learning algorithm (regressor and classifier) was employed for both tasks. To predict the OS, we found the C-index for the baseline radiomics model to be around 0.61 in the discovery set and 0.57 in the validation cohort. With the integrated radiomics-clinical model, the performance of the model improved from 0.61 to 0.63 on the discovery cohort and from 0.57 to 0.62 on the validation cohort. To predict the short-term and long-term survivor groups, the AUC of the radiomics model was found to be 0.64 and 0.58 in the discovery and validation cohorts, respectively. The AUC of the ensemble radiomics-clinical model significantly improved from 0.65 to 0.767 in the discovery dataset and from 0.58 to 0.616 in the validation dataset. The strengths of our study include the large number of patients evaluated in the two institutional cohorts (n = 385) considering the heterogeneity of data across the two hospital centers as well as the use of two clinical endpoints: continuous value of OS and short-term vs long-term survivors.

There can be some challenges with the development of predictive models in a multicenter setting. Firstly, the potential inherent bias in the study design of the retrospective studies arising from multi-institutions was not accounted for in the modeling. Other challenges in the statistical analyses of data include non-standard distributions of measured data, which introduce noisy measurements. This is especially true for high-throughput radiomics-based studies. Introducing new statistical approaches to measure noisy data can improve model predictions, a subject of ongoing investigation. The heterogeneity of scanners across hospitals along with the gray level discretization impacts the radiomics feature extraction. Due to the continuous evolution of scanners, it is possible to have a domain shift in the radiomics feature that occurs as a result of these variations. This can potentially be tackled through the application of harmonization approaches to combat scanner variations [38], which is a topic for future work. Considering robust and reproducible radiomics features extracted from multiple IBSI-compliant radiomics pipelines may potentially improve the model accuracy.

By addressing the above limitations, we hope that the developed radiomics-based prognostic models can be further improved to predict the survival outcomes in advanced NSCLC patients treated with ICIs, which is a subject of ongoing investigation.

In conclusion, we developed parsimonious survival risk models in NSCLC patients treated with immunotherapy, particularly in identifying short- and long-term survivors. This finding may enable the clinicians to design more effective therapeutic regimens or modify treatment strategies for the group of short-term survivors. Nonetheless, additional validation of our models is warranted that could potentially aid in building stable predictive survival models to ICIs.

## Figures and Tables

**Table 1:** Univariate analysis between clinical features and overall survival.

Feature	*p*-Value (OS-R)	*p*-Value (OS Short- and Long-Term Survivors)
Age	0.74255	0.93852
Gender	0.11605	0.13019
ECOG	<10^−6^	0.0039
Smoking status	0.89194	0.93142

## Data Availability

Data presented in this study are not publicly available at this time but may be obtained from the corresponding author, Venkata Manem, upon reasonable request.
